# Audio-Visual Integration in a Redundant Target Paradigm: A Comparison between Rhesus Macaque and Man

**DOI:** 10.3389/fnsys.2017.00089

**Published:** 2017-11-29

**Authors:** Peter Bremen, Rooholla Massoudi, Marc M. Van Wanrooij, A. J. Van Opstal

**Affiliations:** ^1^Department of Biophysics, Donders Institute for Brain, Cognition, and Behaviour, Radboud University Nijmegen, Nijmegen, Netherlands; ^2^Department of Neuroscience, Erasmus Medical Center, Rotterdam, Netherlands; ^3^Department of Physiology, Development and Neuroscience, University of Cambridge, Cambridge, United Kingdom

**Keywords:** auditory, inverse effectiveness, multi-sensory, monkey, non-human primate, operant conditioning, reaction times

## Abstract

The mechanisms underlying multi-sensory interactions are still poorly understood despite considerable progress made since the first neurophysiological recordings of multi-sensory neurons. While the majority of single-cell neurophysiology has been performed in anesthetized or passive-awake laboratory animals, the vast majority of behavioral data stems from studies with human subjects. Interpretation of neurophysiological data implicitly assumes that laboratory animals exhibit perceptual phenomena comparable or identical to those observed in human subjects. To explicitly test this underlying assumption, we here characterized how two rhesus macaques and four humans detect changes in intensity of auditory, visual, and audio-visual stimuli. These intensity changes consisted of a gradual envelope modulation for the sound, and a luminance step for the LED. Subjects had to detect any perceived intensity change as fast as possible. By comparing the monkeys' results with those obtained from the human subjects we found that (1) unimodal reaction times differed across modality, acoustic modulation frequency, and species, (2) the largest facilitation of reaction times with the audio-visual stimuli was observed when stimulus onset asynchronies were such that the unimodal reactions would occur at the same time (response, rather than physical synchrony), and (3) the largest audio-visual reaction-time facilitation was observed when unimodal auditory stimuli were difficult to detect, i.e., at slow unimodal reaction times. We conclude that despite marked unimodal heterogeneity, similar multisensory rules applied to both species. Single-cell neurophysiology in the rhesus macaque may therefore yield valuable insights into the mechanisms governing audio-visual integration that may be informative of the processes taking place in the human brain.

## Introduction

The integration of multi-sensory information benefits object detection, localization, and response latency (e.g., Todd, 1912; Hershenson, 1962; Gielen et al., 1983; Stein and Meredith, 1993; Frens et al., 1995; Corneil and Munoz, 1996; Stein, 1998; Corneil et al., 2002; Van Wanrooij et al., 2009). Deciding which sensory events belong together, i.e., arise from a single object, and which are segregated in time and space necessitate complex sensory processing and decision-making strategies that rely on stimulus statistics (Van Wanrooij et al., 2010; Diederich et al., 2016), cueing (Raab, 1962; Posner, 1980, 2016; Miller, 1982; Spence and Driver, 1996, 1997), reference-frame transformations (Jay and Sparks, 1984; Groh and Sparks, 1992; Goossens and Van Opstal, 2000; Van Grootel et al., 2011), and motivational state (Brosch et al., 2005, 2015). Three basic principles govern multi-sensory integration at neurophysiological and perceptual levels (Stein and Meredith, 1993; Stein and Stanford, 2008; Otto et al., 2013; Stevenson et al., 2014): (1) spatial coincidence (to be integrated, stimuli need to be in close spatial proximity to each other), (2) temporal coincidence (the relative timing of cross-modal stimuli modulates integration), and (3) inverse effectiveness (the strength of multi-sensory integration is inversely related to the efficacy of the unimodal stimuli).

Insights in the neuronal mechanisms underlying these principles stem predominantly from single-unit recordings in anesthetized animals (e.g., Meredith and Stein, 1983; Stein and Wallace, 1996; Bizley et al., 2007; Rowland et al., 2007), from passive awake animals (Schroeder et al., 2001; Bell et al., 2005; Ghazanfar et al., 2005; Schroeder and Foxe, 2005; Kayser et al., 2008), lesion studies in cats (Stein et al., 1989; Burnett et al., 2004; Jiang et al., 2007; Rowland et al., 2014), and from a few combined behavioral and neurophysiological studies in cats (Peck, 1996; Jiang et al., 2002), and non-human primates (Frens and Van Opstal, 1998; Miller et al., 2001; Bell et al., 2005; Fetsch et al., 2011; Brosch et al., 2015; Plakke et al., 2015). Few studies have behaviorally tested multi-sensory perception and decision making in rodents (Sakata et al., 2004; Raposo et al., 2012; Sheppard et al., 2013; Siemann et al., 2015), cats (Jiang et al., 2002), and non-human primates (Bell et al., 2005; Fetsch et al., 2009; Cappe et al., 2010; Brosch et al., 2015; Plakke et al., 2015). In contrast, multi-sensory integration has been tested extensively on the perceptual level in human subjects with a range of paradigms (Spence and Driver, 2003; Koelewijn et al., 2010). Multisensory enhancements in detection and or localization accuracy have been observed in multiple species in different experiments using varying stimuli, task demands, and reward contingencies. Variance in preparation but commonality in results could be interpreted as indicative of some common, basic mechanisms underlying multisensory integration. Nevertheless, in order to link animal neurophysiology and behavior with human psychophysics, it is necessary to directly compare perceptual performance of human and other animal subjects performing in identical tasks.

Here, as a step toward linking perceptual abilities of laboratory animals and humans, we trained two rhesus macaques and instructed four human subjects to perform in an audio-visual paradigm (Raab, 1962; Miller, 1982; Colonius and Diederich, 2012), in which either modality served as the target. We recorded manual reaction times (Donders, 1969; Luce, 1986; Hughes et al., 1994) to the detection of changes in the envelope of a broadband sound, the luminance of a visual stimulus, or to a change in either of the two stimuli. To maximize a potential reaction-time facilitation, the visual and acoustic stimuli were always spatially coincident. We tested various onset asynchronies between the two modalities to determine the influence of temporal coincidence on manual audio-visual reaction times. If temporal coincidence is important, we expect that at a single, fixed onset asynchrony reaction time facilitation is highest. Furthermore, our choice of acoustic stimuli differs from previous studies in one important aspect. To test for inverse effectiveness, we systematically varied the amplitude modulation (AM) frequency of the acoustic stimuli, which is known to have a pronounced effect on unimodal reaction times (Massoudi et al., 2013, 2014). As the luminance change was held constant, we reasoned that if the inverse effectiveness principle applies, audio-visual reaction times should be facilitated most for acoustic modulation frequencies that elicit the slowest reactions.

## Materials and methods

### Ethics statement

All animal experimental procedures complied with the European Communities Council Directive of September 22, 2010 (2010/63/EU) and had been approved by the University's ethics committee for the use of laboratory animals (RU-DEC 2014-049) prior to their execution.

All human psychophysics procedures have been approved by the local ethics committee of the Faculty of Social Sciences of the Radboud University (ECSW, 2016), as they concerned non-invasive observational experiments with healthy adult human subjects. Prior to their participation in the experiments, volunteers gave their written informed consent.

### Subjects

Two male rhesus monkeys (Macaca mulatta) aged 10 (M1) and 6 (M2) years, and four human listeners (H1 to H4; H4 female) aged between 34 and 37 years, participated in our experiments. Monkey M1 had participated in an earlier unpublished and unrelated auditory detection experiment from our laboratory while monkey M2 was completely naïve to all procedures. The two monkeys were pair-housed with each other, and monkey M1 was the alpha male. Housing included cage enrichment in the form of wooden climbing rods, car tires, hammocks, daily food-puzzles (puzzle balls, custom-made sliding trays, seeds thrown on the floor bedding material), and video screening (thrice weekly for 1 h). To provide the animals with a stimulating environment and to prevent monotony we changed cage enrichment regularly on a predetermined schedule. We changed the food-puzzles on a daily basis and rearranged cage interior on a bi-weekly basis. Two listeners (H3 and H4) were naïve as to the purpose of the study, while listeners H1 and H2 are authors of this paper. All listeners had normal audiometric hearing within 20 dB of audiometric zero. Listeners H1 and H2 wore their prescription glasses during the experiments. We did not specifically assess visual acuity and hearing thresholds in the two monkeys. However, the animals were capable of detecting changes in both luminance and the temporal envelopes of broadband sounds over a wide range of stimulus parameters.

### Stimuli

We created amplitude-modulated noise of duration D that allowed us to manipulate the envelope of the noise. The noise, *S*(*t*), consisted of a static part of variable duration D_S_ and an amplitude-modulated part of duration D_AM_ = 1 second (Massoudi et al., 2013). *S*(*t*) comprised 128 simultaneously presented tones ranging from *f*_*0*_ = 250 Hz to *f*_*127*_ = 20.4 kHz in 1/20-oct steps. For the *f*_*0*_ component we fixed the phase Φ_*0*_ at π/2, and for all other components we randomized the phase, Φ_*n*_, between –π and +π.

(1) $$S(t)=\sum_{n=1}^{128}R(t)\cdot \sin(2\pi \cdot f_{n}\cdot t+\phi_{n})\;for\;-\pi <\;\phi_{\text{n}}\;<\;+\pi$$

with

$$f_{n}=f_{0}\cdot 2\frac{\left(n-1\right)}{20}\;for\;1\;<\;n\;\leq \;128$$

We modulated the noise with a sinusoidal envelope such that:

(2) $$\begin{array}{l} R(t)=1\;for\;0\;\leq \text{ t }\leq \;{\text{D}}_{\text{S}}\\ R(t)=1+\Delta M\cdot \sin(2\pi \omega t)\;for\;{\text{D}}_{\text{S}}\;<\text{ t }\leq \text{ D}. \end{array}$$

t indicates time re. sound onset (in s); ω is the temporal modulation rate (in Hz); ΔM is the modulation-depth on a linear scale from 0 to 1, which was fixed for the audio-visual paradigm at 0.25; D_S_ defines the duration of the static noise (ω set to zero) at the onset of the stimulus sequence. In the modulated part of the stimulus (*t* > D_s_), ω ranged from 2 to 256 Hz on a binary logarithmic scale. Additionally, to avoid click artifacts we ramped the onset and offset of *S*(*t*) with 5-ms sin^2^/cos^2^ ramps.

We generated all stimuli digitally at 48828.125 Hz with 24-bit precision using System 3 equipment from Tucker-Davis Technologies (RZ6, TDT, Alachua, FL), and played the sounds via an oval two-way speaker (SC5.9, Visaton GmbH, Haan, Germany). We used a precision microphone (model no. 7012; ACO Pacific, Belmont, CA) positioned at the location of the subject's head to calibrate the speaker by producing a look-up table for pure tones from 0.2 to 18.1 kHz in 1/6-oct steps. We presented all stimuli at a sound level of 50 dB SPL.

For visual stimulation, we used a 5-mm diameter, bi-color red (λ_red_ = 617 nm) and green (λ_green_ = 568 nm) LED (L-59EGW, Kingbright Electronic, Co., Ltd.) mounted in front of the speaker and centered in the speaker-box. We controlled the LED via an Arduino Uno board (www.arduino.cc) that received color, intensity, and timing information from the TDT real-time processor used to generate the sounds. We calibrated the LED for all colors used with a precision luminance meter (LS-100; Konica Minolta Sensing, Singapore) positioned at the location of the subject's eyes and aligning the LED with the center of the light sensor of the instrument. In that way, we created a look-up table for all luminance values used in the present study.

Our choice of auditory stimuli was motivated by (a) the importance of amplitude modulation for speech processing, and (b) the suitability for future neurophysiological experiments. We chose dimming of the LED (see below) because (a) a step-like small change in luminance requires the subject to focus on the task in order not to miss the change, and (b) non-human primates can be trained readily to detect luminance changes; a paradigm widely used e.g., in oculomotor research. We chose the used stimulus parameters based on pilot experiments. We wanted to include stimuli that most likely (a) maximize multisensory benefit, (b) minimize benefit, and (c) fall in-between these two extremes.

### Procedure

Experiments were conducted in a completely dark, sound-attenuated room (3 × 3 × 3 m) located in the animal housing facility and lined with sound-absorbent foam (50 mm thick with 30 mm pyramids, AX2250, Uxem b.v., Lelystad, The Netherlands). The room had an ambient background noise level of ~30 dB SPL and no reverberations for frequencies above 500 Hz. The speaker and LED were positioned at the height of the subject's inter-aural axis straight ahead at a distance of 1.25 m. All experiments were performed with head-unrestrained subjects and without the use of quantitative head- or eye-tracking techniques. We monitored the subjects via an infrared-surveillance camera and observed that the subjects were looking straight ahead when engaged in the task. The monkeys typically aligned their pinnae with the straight ahead sound source.

Human listeners sat on a straight-back chair and were instructed to keep their head still and to fixate the LED with their eyes during stimulus presentation. Monkeys sat in a custom-made primate chair that doubled as a transport cart. Although the primate chair did introduce slight frequency-specific distortions of the sound field, we deem these distortions of no perceptual significance. We trained the monkeys and instructed the human subjects to break an infrared beam with their right hand to initiate a trial and to remove the hand upon detection of (1) a change from unmodulated to amplitude modulated noise, and (2) a change of intensity of the LED. After training, the animals were able to perform in five different paradigms: (1) unimodal auditory, (2) unimodal visual, (3) audio-visual redundant target (RTP), (4) auditory-cued audio-visual focused attention (red cue LED), and (5) visually-cued audio-visual focused attention (green cue LED). Here, we report on data from the first three paradigms. In the present context it is noteworthy that we first collected all data for the two focused attention paradigms before training the animals on the RTP. We based our decision to do so on the following assumptions. The RTP is less challenging than the focused attention paradigms. It does not require to withhold a response to the non-cued modality. Motivational issues could arise if the two classes of audio-visual paradigms were to be randomly interleaved within a block or session. In all sessions, however, we pseudo-randomly included unimodal auditory and visual blocks to strengthen the association between the colors red and green with auditory and visual trials, respectively.

By participating in the experiments the monkeys earned water rewards for a minimum of 20 ml/kg/day. In order to guarantee that the monkeys drank the required minimum amount of water we adjusted the reward size on a trial-by-trial basis as necessary. With this strategy, we were able to ascertain that the animals maintained homeostasis without supplemental administration of water. After successful training animals would regularly drink more than the required daily minimum of 20 ml/kg. Especially the older animal (M1) could occasionally reach 30–40 ml/kg/day without noticeable differences in performance on the subsequent day. Daily records were kept of the monkeys' weight, water intake, urine density/refraction, and general health status. All of these parameters stayed well within the physiological healthy range during the course of the experiments. After the behavioral session, we supplemented the dry food rations available in the cages with various sorts of vegetables and fruit.

We trained the monkeys in a stepwise manner. First, the animals learned to detect the auditory change. For that we used stimuli with short static-noise intervals. We systematically increased the intervals until we achieved the range used in the experiments. After performance was equal or better to 80% correct on three consecutive days, we generalized the animal on all modulation frequencies. After generalization we switched to the visual task and repeated the incremental increase of the static stimulus period until the experimental range was reached. Since the basic task structure remained the same both animals quickly reached the criterion of 80% performance on three consecutive days with the visual stimulus. After generalizing, i.e., testing various luminance differences, we interleaved auditory and visual trials within one block. Next, we introduced cued focused attention audiovisual trials (paradigms 4 and 5). After training and completion of data collection for the focused attention paradigms, we introduced the RTP. Upon reaching 80% correct in three consecutive session we began to collect the data presented here. In contrast, the human subjects were verbally informed about the tasks and the associated LED color cues. We instructed them “to react as quickly and accurately as possible upon detection of the cued stimulus change.” All human subjects performed one training block to (a) accustom the subject to the general task structure, and (b) verify that the subjects were capable of performing the task adequately.

### Detection paradigms

**Figure 1:**
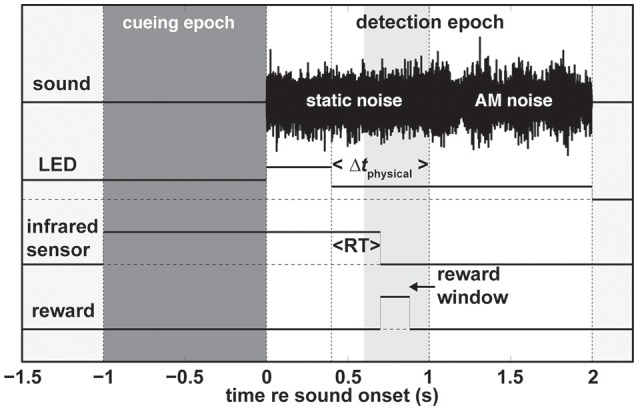
General trial structure. A trial consists of two epochs (1) the cueing epoch (dark gray patch), and (2) the detection epoch (white patch). In this particular example of a redundant target trial the change in LED intensity occurs earlier than the sound change (Δt_physical_) and is rewarded if the subject releases the infrared sensor (at reaction time RT) within the reward window (light gray patch). For unimodal auditory and visual trials during the detection epoch the LED and static noise would not change, respectively. Note that in the case of the human subjects there was no reward window and we used different modulation frequencies for humans and monkeys. Refer to the main text for more details. AM, amplitude modulated.

We used the following basic paradigm structure (Figure 1). To signal to the subject that a trial was ready the LED turned on. The program than waited for the subject to activate the infrared-sensor button and the LED would stay on for another 1,000 ms; the cueing epoch (dark gray patch in Figure 1). The color of the LED in this initial epoch served to cue the subject on which modality to pay attention to (Table 1). In the unimodal paradigms, red and green indicated that the change of the sound and LED intensity, respectively, should be reported by releasing the infrared-sensor button. In the audio and visual unimodal paradigms either the sound would become amplitude-modulated or the LED would dim after a random time interval (400/1,000–2,000 ms in steps of 200 ms for monkeys/humans) while the other modality would not change. That is, the LED would not dim in the unimodal audio paradigm, and the sound would not become amplitude modulated in the unimodal visual paradigm. For both monkeys and humans in all audio-visual paradigms the random time interval was 1,000–2,000 ms in steps of 200 ms to accommodate the temporal shifting of the non-cued modality (parameter Δt_physical_). In the redundant target paradigm (RTP), cued by the color orange, both modalities changed and the first such change irrespective of modality should be detected. Note that during the detection epoch (white patch in Figure 1) starting at the beginning of sound onset the intensity of the LED increased.

**Table 1:** LED color of the 1,000-ms cueing epoch at the beginning of a trial and the trial proper following the initiation of the sound for the various unimodal and audio-visual paradigms.

**Paradigm**	**LED color during cueing epoch**	**LED color during detection epoch**
Unimodal auditory	Red	Orange
Unimodal visual	Green	Orange
Audio-visual redundant target	Orange	Orange

For all paradigms, we ensured the monkey's vigilance by implementing a reward window (light gray patch in Figure 1) between 200 and 600 ms after the cued change. Additionally, we enforced a time-out period of 5 s if the monkey either released the infrared sensor prior to the cued change or if it did not release the sensor at all during a trial. We did not reward, punish, or provide any type of feedback to the human subjects. The sound and the LED were extinguished as soon as the subject released the infrared sensor irrespective of the epoch and current time.

To test the influence of the temporal delay between the leading and lagging modalities on reaction times (RTs) we systematically varied the onset difference between the leading and lagging modalities. We used the following timing differences Δt_physical_ = {±600, ±400, ±200, ±75, 0} ms with negative values indicating that the visual modality was leading. All parameter combinations were presented randomly within a block. For example, a block of the audio-visual RTP would contain randomly presented audio-change leading and visual-change leading trials with all of the Δt's repeated three times for a total of 81 trials. We pseudo-randomly interleaved pure unimodal blocks (either visual or auditory) with audio-visual blocks within one session and across sessions. The animals would complete between 400 and 1,200 trials per day. Note that the Δt_physical_ = ±600 ms conditions (depending on the modality) are effectively unimodal conditions for RTs < 600 ms; this holds also for trials in which the subject reacts prior to the change of the lagging modality.

### Data analysis

**Figure 2:**
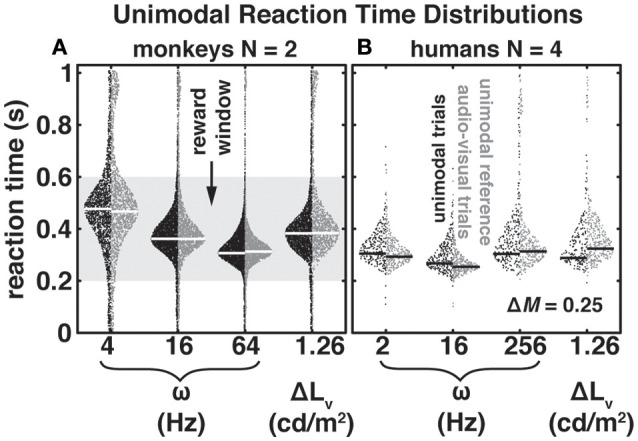
Bee-swarm plots for unimodal (black) and audio-visual (dark gray) reaction times (RTs) as a function of modulation frequency, ω, and luminance difference, ΔL_v_, for two monkeys **(A)** and four humans **(B)**. In the auditory trials modulation depth, Δ*M*, was 0.25. For auditory and visual audio-visual RTs we selected data in which either the auditory or visual change led by 600 ms. These conditions can be considered “unimodal” since the far majority of RTs was < 600 ms for all stimuli. Dots indicate individual RTs and white (monkeys) and black (humans) horizontal lines the mode of the distributions. Note that in contrast to the monkeys, humans were not rewarded and did not receive feedback for responding within the reward window (gray patch in **A)**.

**Figure 3:**
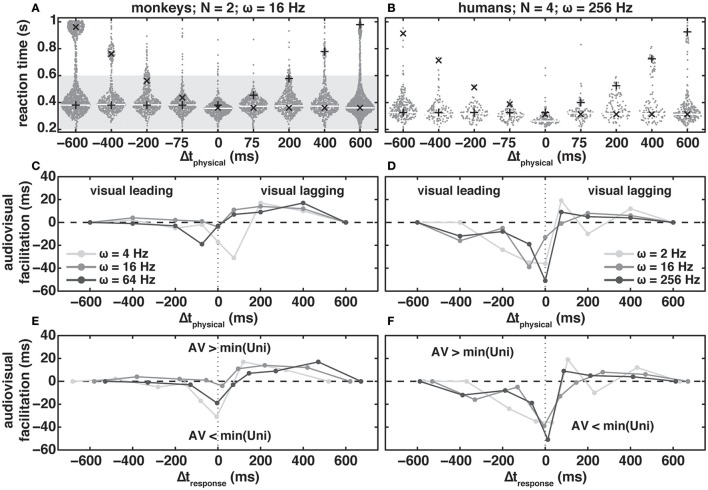
Bee-swarm plots **(A,B)** for audio-visual reaction times (RTs) in the redundant target paradigm as a function of modulation frequency, ω, for two monkeys **(A)** and four humans **(B)**. Modulation depth, Δ*M*, and luminance difference, Δ*L*_v_, were 25% and 1.26 cd/m^2^, respectively. Dots indicate individual RTs and the white (monkeys) and light gray (humans) horizontal lines the mode of the audio-visual distribution. Black “pluses” and “crosses” indicate the mode of visually and auditory evoked RTs in the Δt = −600 ms and Δt = 600 ms conditions, respectively, shifted by Δt_physical_. Note that in contrast to the monkeys, humans were not rewarded and did not receive feedback for responding within the reward window (dark gray patch in **(A)**. Audio-visual facilitation as a function of physical **(C,D)** and response **(E,F)** delay for monkeys **(C,E)** and human subjects **(D,F)** at three different amplitude-modulation frequencies (shades of gray). Negative and positive values, respectively, indicate a speed-up/decrease and a slow-down/increase of reaction times re. fasted unimodal response. For details see main text.

We implemented all data analysis in Matlab R2012a (The Mathworks Inc.). To provide an overview of the raw RTs and the underlying distributions we display the data as bee-swarm plots (Figures 2, 3): In such plots, we approximated a given empirical RT distribution by employing a kernel-smoothing density-estimation procedure (from −3 to +1 s in steps of 1 ms with the Matlab function “ksdensity” assuming an unbounded normal distribution). We then pseudo-randomly plotted the RTs within the boundaries of the estimated and mirrored (re. abscissa) distribution. The mode of the reaction time distributions was taken as the most-probable reaction time as determined by this density-estimation procedure. We determined the mode, rather than the typically-used mean, as in certain conditions bimodal RT distributions were elicited due to subjects missing the leading stimulus and responding to the lagging stimulus. The mode represents the reaction time value that appears most often in the estimated distribution. Since most of the responses occur to the leading stimulus change, the mode results in a more stable and representative estimate.

To quantify a subject's motivation we calculated the early release, or lapse, rate. We defined the early infrared-sensor release rate, *RR*_*early*_, as follows:

(3) $$RR_{early}=\frac{N_{pre}}{N_{pre}+N_{post}}\cdot 100,$$

with *N*_*pre*_ the number of reaction times (RTs) falling in the interval −1,000 ≥ RT ≤ 0 ms, and *N*_*post*_ the number of RTs falling in the interval 0 ≤ RT ≤ 1,000 ms. Animal subjects performing in detection paradigms exhibit lapse rates of about 20% depending on task difficulty (Penner, 1995; O'Connor et al., 2000).

We calculated the audio-visual facilitation, *F*, for the redundant target paradigm as

(4) $$F=RT_{AV}-\text{min}([RT_{A},RT_{V}]),$$

with *RT*_*AV*_ the audio-visual RT mode in the RTP, *RT*_*A*_, and *RT*_*V*_ the RT modes of the RTP in the Δt_physical_ +600 ms, i.e., auditory change leading, and −600 ms, i.e., visual change leading, conditions, respectively. Our rationale for this approach was to exclude context effects between the unimodal and audio-visual blocks. Since the reaction time modes for the +600 and −600 ms conditions turn out to be <600 (e.g., Figure 2), these modes can be considered to be elicited only by the leading unimodal stimulus. Negative facilitation values indicate a speed-up of RT re. unimodal RT while positive values indicate a slow-down of the response (for alternative measures of facilitation see Colonius and Diederich, 2017). To account for the neural sensorimotor processing time, we correct the physically applied timing delay, Δt_physical_, by adding the difference *RT*_*A*_–*RT*_*V*_ modes to Δt_physical_ to obtain the timing delay accounting for auditory and visual response time differences, Δt_response_ (see Figure 3). In this way, we corrected for the modality specific internal processing time.

Since it is known that RT in auditory detection paradigms depends systematically on modulation frequency ω (e.g., Massoudi et al., 2013, 2014), we expected ω to modulate audio-visual facilitation. Modulation frequencies that lead to slow unimodal RTs should result in more audio-visual facilitation, when compared to modulation frequencies eliciting faster unimodal responses. Therefore, to explicitly test the inverse effectiveness principle we analyzed audio-visual facilitation, *F*, as a function of *RT*_*A*_. However, to correct for subject-specific differences in RT (slow vs. fast responders), we subtracted for each modulation frequency the subject's fastest observed RT across all audio-visual stimulus conditions, *RT*_*fast*_, from their *RT*_*A*_ to obtain *RT*_*corrected*_. To quantify inverse effectiveness, we then performed a linear regression analysis:

(5) $$F={\text{a}}^{*}RT_{corrected}+\text{b},$$

with a (dimensionless) and b (in ms) the slope and offset of the optimal regression line, respectively. Parameters were found by minimizing the mean squared error (Press et al., 1992).

To statistically compare RT distributions with each other we used the non-parametric Kolmogorov-Smirnov test with an alpha, α, of 0.01 and applied the Bonferroni-Holm correction when comparing multiple distributions with each other. Where appropriate we performed N-way analysis of variance (ANOVA) with interaction terms.

## Results

### Data base

Monkeys M1 and M2 completed a total of 37,090 and 36,249 trials in 65 and 93 sessions, respectively. The four human subjects completed 5,052 (H1), 2,492 (H2), 1,401 (H3), and 1,360 (H4) trials in 17 (H1), 11 (H2), 4 (H3), and 5 (H4) sessions. Initially, we conducted all analyses on a per subject basis. For the monkeys we did not observe obvious intra-subject differences across frequencies [*F*_(df = 2)_ = 4.99, *p* = 0.155; N-way ANOVA]. For human subjects we did find idiosyncratic differences in absolute reaction times [*F*_(df = 2)_ = 10.85, *p* = 0.007; N-way ANOVA]. Since we were not interested in idiosyncratic inter-subject differences we pooled data within the two species for all subsequent analysis.

### Unimodal responses

In the unimodal audio paradigm, we varied the modulation frequency (temporal modulation rate), ω, of the noise, and held modulation depth, Δ*M*, constant. Note that we randomly interleaved unimodal audio and visual trials within a block of a session. First, we observed that the mean early infrared-sensor release rates, *RR*_*early*_ (RTs < 0 ms), across all change times, t_c_, and conditions were ~10 and ~15% for monkeys M1 and M2, respectively. These values are in good agreement with lapse rates previously reported for animals performing in unimodal detection paradigms (Penner, 1995; O'Connor et al., 2000). Averaged across change times and stimulus parameters, early-release RTs peaked between 300 and 400 ms prior to the stimulus change for both monkeys. For the four human listeners *RR*_*early*_‘s were ~1% (H1), ~1% (H2), ~5% (H3), and ~1% (H4) and thus much lower than those observed in the monkeys. Due to the extremely low *RR*_*early*_‘s in human listeners it was not feasible to estimate their early-release RT distributions.

In Figure 2 we present RTs as a function of modulation frequency, ω, with a fixed modulation depth, Δ*M* = 0.25. To provide an impression of both the raw data and the distributions we present the data in the form of bee-swarm plots. This type of plot distributes individual data points (dots) pseudo-randomly within the boundaries of their underlying distribution (see Methods). Note that for a given parameter the left side depicts RTs from the unimodal condition (black) and the distributions on the right side the corresponding RT distribution from a matched audio-visual condition (dark gray). We indicate the mode of the distributions with thick white and black horizontal lines for monkeys and humans, respectively. To reward the monkeys, but not the humans, during data collection we applied a “reward” window (gray patch); this window size leads to a 40% guess rate.

Note that both in monkeys and humans RTs changed as a function of modulation frequency [monkeys: *F*_(df = 2)_ = 34.76, *p* = 0.028; humans: *F*_(df = 2)_ = 15.4, *p* = 0.003; N-way ANOVA]. The differences between adjacent modulation frequencies were in all cases about 50 ms. However, while the RTs of the human listeners (Figure 2B) changed only slightly at the lowest and highest modulation frequencies shown here, monkey RTs (Figure 2A) systematically decreased with increasing modulation frequency. This observation is in good congruence with previous findings based on threshold measurements and d'-analyses (Moody, 1994; O'Connor et al., 2011) demonstrating that monkeys are less sensitive to slow modulations compared to humans. While peak modulation frequency sensitivity of the rhesus macaque lies roughly between 30 and 300 Hz, sensitivity in the human peaks between 4 and 100 Hz.

Figure 2 also shows the unimodal LED-dimming reaction time distributions to the luminance value used in the audio-visual paradigms. In the monkeys (Figure 2A) visually-evoked RT-distribution modes were slower than auditory-evoked RTs for ω = 16 and 64 Hz, but faster than the mode for ω = 4 Hz. In the humans (Figure 2B) visually-evoked RTs fell in-between the RTs for ω = 16 Hz and ω = 2 and 256 Hz and were not significantly different from ω = 2 Hz (Kolmogorov-Smirnov test with Bonferroni-Holm correction for multiple comparisons; p = 0.19). Visual RT distributions of monkeys and humans were significantly different from each other (Kolmogorov-Smirnov test; *p* << 0.01). Similar to what we found for audio-evoked RTs early infrared-sensor release rates, *RR*_*early*_ (RTs < 0 ms), in the visual paradigm were ~16% and ~20% for monkeys M1 and M2, respectively. Those rates were somewhat higher than the ones observed for audio trials (see above). Although within the span of reported lapse rates for animals (Penner, 1995; O'Connor et al., 2000) this slight discrepancy may be due to the step-like nature of the luminance change, which rendered the visual stimulus more difficult to detect than the ongoing change in the audio stimulus. Like for the audio trials, the visual *RR*_*early*_ distributions peaked between 300 and 400 ms prior to the stimulus change for both monkeys. Early release rates for the human subjects remained low at ~1% (H1), ~1% (H2), ~2% (H3), and ~0% (H4).

We expected to find potential context effects when presenting purely unimodal stimuli embedded in an audio-visual block of trials, which could lead to a constant RT offset, i.e., either a speeding or slowing of RTs (Burr and Alais, 2006). Therefore, in the audio-visual paradigm we included trials with a delay between the leading and lagging modality that exceeded the slowest RTs of the subjects. Specifically, in these conditions, the auditory or the visual change could lead the other modality by 600 ms. Accordingly, RTs in these audio-visual stimulus conditions can be assumed as being elicited by the leading modality. These trials served as unimodal references in the audio-visual context. The RTs for these audio-visual timing conditions are shown with dark gray dots in Figure 2. The monkeys did not exhibit strong systematic context effects; compare black with dark gray dots per stimulus in Figure 2A. We observed no difference in RT mode for 16 Hz and only minor non-systematic mode differences of 10 and −6 ms, for ω = 4 and 64 Hz and −1 ms with the visual stimulus; negative values indicate longer RTs in the audio-visual condition. Human subjects (Figure 2B) exhibited more obvious RT-mode differences that were especially pronounced in the visual condition. The RT-mode difference in that condition was −36 ms, i.e., the RT to a visual change in an audio-visual context was slower re. unimodal context. RTs to auditory changes in an audio-visual context could be slightly faster (2 Hz: 12 ms; 16 Hz: 13 ms) and slightly slower (256 Hz: −9 ms) re. unimodal context. Due to the differences of the human RTs in unimodal and audio-visual contexts we opted to use the unimodal RTs obtained in an audio-visual context as a reference for RT normalization (see Methods).

As described in the Introduction, the principle of inverse effectiveness states that multi-sensory integration is strong if unimodal stimuli are weak, i.e., lead to a high degree of perceptual uncertainty (Stein, 1998; Corneil et al., 2002; Bell et al., 2005; Van Wanrooij et al., 2009). The auditory and visual stimuli selected in the current experiments, namely ω = {4, 16, 64} Hz and ω = {2, 16, 256} Hz for the monkeys and humans, respectively, and a luminance change of 1.26 cd/m^2^ were well-suited to test the inverse effectiveness principle. The modulation frequencies used for the monkeys could be interpreted as perceptually weak (4 Hz), moderate (16 Hz), and strong (64 Hz). Due to the U-shaped curve in the human subjects our selection was limited to two moderately difficult stimuli (2 and 256 Hz) and one easily detectable stimulus (16 Hz). The latter was also employed with the monkeys. In both monkeys and humans the luminance change elicited RTs roughly in-between the fastest and slowest auditory RTs, and may therefore be expected to interact with auditory processing. Taken together, our unimodal data from both the unimodal and audio-visual contexts were characterized by a good heterogeneity of RTs across modalities, auditory modulation frequencies, and species.

### Redundant target paradigm (RTP)

In audio-visual conditions, subjects were required to react to the first stimulus change, irrespective of modality. Figures 3A,B show RTs (dark-gray dots) obtained in the RTP as a function of the physical delay between the changes of the auditory and visual stimuli, Δt_physical_, for two monkeys at ω = 16 Hz (Figure 3A), and four human subjects at ω = 256 Hz (Figure 3B). Thick white (monkeys) and light-gray (humans) lines indicate the modes of the RT distributions, while crosses and pluses indicate the modes of the “unimodal” auditory and visual distributions obtained from the Δt +600 ms and −600 ms conditions of the RTP, respectively; the unimodal mode predictions have been corrected for the applied Δt_physical_ (see Methods).

When focusing on responses within the range of Δt_physical_ = 200–600 ms, it appears that audio-visual RTs for the monkeys (Figure 3A) largely followed the “unimodal” predictions. That is, if the visual change was leading, Δt_physical_ < 0 ms, RT modes in the RTP aligned closely with the unimodal modes (crosses). For humans (Figure 3B), RT modes in the RTP were slightly faster than the unimodal predictions. In both monkeys and humans, we observed a slowing of RTs with Δt_physical_ > 0 ms, i.e., the auditory change led the visual one. The strongest positive interaction of auditory and visual stimulus changes in the form of a decrease in RT occurred for Δt_physical_ = 0 ms in both species. With physically coinciding changes the speeding of RT re. fastest unimodal RT was 5 and 51 ms for monkeys and humans, respectively. Note that for this timing condition the unimodal RT modes aligned closest compared to all Δt-conditions. Furthermore, monkeys and humans both reacted to the lagging modality. These incorrect responses falling (800 < RT < 1,000 ms) are most conspicuous for the monkeys at Δt = −600 ms and account for roughly 20% of responses across the interval 0 to 1,000 ms (the distribution is not sampled in its entirety because recording ended 1,000 ms after the first stimulus change). In contrast, the percentage of responses to the lagging visual change is only 7% in the Δt = +600 ms timing condition. This difference is most likely attributable to the step-like change in the visual dimming, in contrast to the ongoing amplitude modulation of the auditory stimulus. Note also that with a modulation frequency of 4 Hz the percentage of lagging responses was 1% for both visual and auditory lagging changes, while with 64 Hz these percentages were 0.4 and 30%. This stimulus-dependence of the percentage of responses to the lagging change seems to suggest that the monkeys were performing the task but sometimes missed the leading change. Overall the human subjects made fewer responses to the lagging change, which nevertheless could exert a strong influence on the RT distribution (Δt_physical_ = +200 ms).

In Figures 3C,D we quantified the RT facilitation (see Methods) of audio-visual change detection for all modulation frequencies tested in the monkeys (Figure 3C) and humans (Figure 3D), plotted as a function of physical delay, Δt_physical_. Negative facilitation indicates a speeding of audio-visual responses re. the fastest unimodal RT mode (labeled min(Uni) in panels E and F of the figure), while positive values indicate a slowing of the audio-visual RTs (see Methods). In congruence with the existence of a time window of integration, in both monkeys and humans we found a statistically significant effect of Δt_physical_ on RT [monkeys: *T*_(df = 6)_ = 7.79, *p* = 0.012; humans: *T*_(df = 6)_ = 13.17, *p* = 0; repeated measures ANOVA]. In the case of the monkeys we did not observe an effect of modulation frequency on RT [*T*_(df = 6)_ = 0.49, *p* = 0.671; repeated measures ANOVA]. In contrast, in humans there was a significant effect [*T*_(df = 6)_ = 18.45, *p* = 0.002; repeated measures ANOVA] such that = 16 Hz resulted in the least amount of audiovisual facilitation. In general, we found that both monkeys and humans exhibited strong interactions of the two modalities. Facilitation predominantly occurred with stimuli in which Δt_physical_ is ≤ +75 ms, i.e., the visual change leads or lags only by a small interval, while responses are slowed down for auditory leading stimulus conditions. Note that (1) the interaction effects are stronger in the humans than in the monkeys, and (2) occur over a larger range of Δt‘s in humans. In addition, a maximum speed-up across modulation frequency occurred at different physical delays for the two species. For example, in the monkeys (Figure 3C) maximum RT speed-up with a modulation frequency of 4 Hz occurs at Δt_physical_ = +75 ms and at 0 ms, and at −75 ms for 16 Hz and 64 Hz, respectively. A qualitatively similar variation can be observed for the human subjects (Figure 3D). This modulation-frequency-dependent shift in maximum facilitation can be accounted for by incorporating the unimodal auditory RT differences. In Figures 3E,F we corrected the physical stimulus delay, by adding the difference between the unimodal visual and auditory RT modes to calculate Δt_response_ (see Methods). After this correction, maximum facilitation occurred at or close to 0 ms, and the curves for the individual modulation frequencies aligned more closely. Nevertheless, the amount of facilitation remained modulation-frequency dependent. We found the largest RT facilitation at ω = 4 Hz in the monkeys (−33 ms), and at ω = 256 Hz in the humans (−51 ms). In the human subjects, modulation frequencies of 2 and 16 Hz led to a comparable facilitation. In contrast, the facilitation differences between ω = 16 Hz (−5 ms) and 64 Hz (−19 ms) in the monkeys were unexpected given the observed unimodal auditory RTs differences for these modulation frequencies, and their implications for inverse effectiveness in audio-visual integration. From the RT distributions (Figure 2) one would expect least facilitation at ω = 64 Hz. This was, however, not the case. Instead we obtained least facilitation for ω = 16 Hz.

To better understand this apparent discrepancy with regard to inverse effectiveness, we performed the following analysis. We argued that facilitation should be inversely correlated with amplitude-modulation detection difficulty, as obtained from unimodal RT modes. However, to compare RTs across subjects, it is necessary to correct for idiosyncratic variability of RTs (overall slow responders vs. fast responders). We achieved this by subtracting per subject the fastest RT mode across all audio-visual conditions from the unimodal RT mode, RT_A_. Positive (negative) corrected RTs then indicate that the unimodal RT mode is slower (faster) than the fastest audio-visual RT mode. If the inverse effectiveness principle holds true, one expects a negative correlation between facilitation and corrected reaction times. For instance, if the unimodal auditory stimulus was difficult to detect (large positive corrected RTs) facilitation should be large (large negative corrected reaction times).

**Figure 4:**
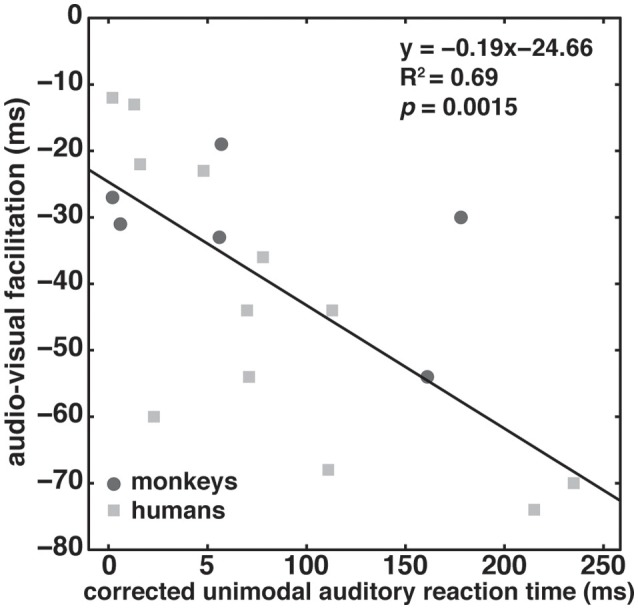
Inverse effectiveness. Audio-visual facilitation as a function of corrected unimodal auditory reaction times corrected for an individual subject's overall response speed (see section Materials and Methods). Data from the two monkeys and four humans are shown as dark gray circles and light gray squares, respectively. Each subject contributed three data points based on the three tested auditory modulation frequencies. The black line indicates a linear fit through the pooled data of both monkeys and humans.

Figure 4 depicts the strength of audio-visual facilitation as a function of corrected unimodal auditory RT for the two monkeys (dark gray circles) and the four human subjects (light gray squares); each subject contributed three data points based on the three tested modulation frequencies. The data could indeed be described with a significant (*p* = 0.0015) linear regression line with a negative slope (−0.19), and a high correlation coefficient (*R*^2^ = 0.69). The intercept at facilitation of 25 ms suggests that even when the corrected RT is 0 ms, facilitation still occurs. We conclude that the inverse effectiveness principle holds true for both humans and monkeys.

## Discussion

This study shows that despite a pronounced heterogeneity of unimodal reaction times across modality, acoustic modulation frequency and species, multisensory rules applied to audio-visual reaction times of rhesus macaques and humans. Particularly, reaction time facilitation in an audio-visual context was related to (1) the timing of the auditory and visual stimuli, and (2) the perceptual detectability (quantified by the modes of unimodal reaction-time distributions). With regard to the temporal congruency principle, we indeed observed the largest facilitatory effects of reaction times in conditions for which auditory and visual sensorimotor processing were synchronized. Also, the inverse effectiveness principle held true in that audio-visual facilitation correlated negatively with unimodal auditory reaction times; these varied systematically with the detectability of amplitude-modulated broadband noise (Figure 4). Despite quantitative differences, similar rules of temporal congruency and inverse effectiveness applied (Otto et al., 2013) to both species. We therefore consider macaques an excellent animal species to study the neuronal mechanisms involved in multisensory integration. Additionally, our finding underlines the validity of comparative neurophysiological experiments that aim to unravel the neuronal mechanisms subserving multisensory integration in animals including humans. Furthermore, we note that reductionist stimuli, such as amplitude-modulated noise and a dimming light, can elicit true audio-visual integration. This point is important since such stimuli allow for the study of audio-visual integration in well-controlled parameterized paradigms that nevertheless capture features of more realistic every-day situations such as speech understanding in crowded environments or with a hearing impairment. Such paradigms can be used readily in single-cell neurophysiological experiments with laboratory animals. We would therefore like to propose that in the design of multisensory studies aimed at maximizing the visibility of multisensory effects particular attention should be payed to selecting the parameters of the unimodal stimuli such that interactions are facilitated (Miller et al., 2015, 2017). As we have demonstrated here, amplitude modulated sounds are a promising candidate for this.

### Comparison between macaque and man

The main aim of our study was to directly compare rhesus macaques and humans in the same audio-visual paradigm to assess possible species differences and similarities. As outlined in the introduction section it is typically assumed that basic mechanisms underlying multisensory integration are independent of species, task engagement, and for laboratory animals anesthesia. Ultimately, this assumption needs to be tested by obtaining behavioral data that allow for the interpretation of neurophysiological data obtained from anesthetized or passive animals. Our data suggest that the assumption is valid in that compared to human subjects the rhesus macaque exhibits similar audio-visual integration effects despite the marked differences in unimodal processing between the two species summarized below.

The most obvious difference between monkeys and humans is in their auditory temporal processing sensitivity (Figure 2). While macaques are most sensitive to high temporal modulations between ~5 ≤ ω ≤ 500 Hz with a peak between 30 and 250 Hz that depends on sound duration (O'Connor et al., 2011), humans are most sensitive to slow temporal modulations up to a few 100 Hz with a peak at about 10 Hz (O'Connor et al., 2011). These species differences are mirrored in the prevalent temporal modulations in monkey and human vocalizations, and are supposedly an adaptation to the respective ethological niche (Cohen et al., 2007).

Detection of luminance changes is very comparable between monkeys and humans (Figure 2). We noticed that in our interleaved unimodal auditory and visual paradigms monkeys exhibited longer reaction times given the same luminance change when compared to the human subjects. We speculate that this discrepancy may be due to our choice of reward contingency for the monkeys. The relatively small window width of 400 ms opening 200 ms post change onset may have led the animals to adopt a conservative response strategy, i.e., avoiding very fast responses, in order to maximize reward output. We did not impose such a window for the human subjects and additionally instructed them to respond as quickly as possible. We did not test this hypothesis directly but note that reward conditions have been shown to modulate an animal's response behavior (Brosch et al., 2005; Schultz, 2006).

Despite these differences in unimodal reactions times, bimodal reaction times in both species exhibited very similar audio-visual effects (Figures 3, 4). We found maximal facilitation when the unimodal responses coincided temporally (Δt_response_ near 0 ms, Figures 3E,F). Facilitation decreased when temporal coincidence of the responses decreased. Inhibition occurred when the auditory response would be faster on average than the visual response. Human subjects did seem to integrate over a larger temporal window than monkeys.

### Interpretation of inverse effectiveness principle

Similarly, we found that both monkey and human reaction times conformed to the inverse effectiveness principle of multi-sensory integration. One may expect that any observed audio-visual effect may be inversely related to the ease with which the unimodal stimuli can be detected (Miller et al., 2015). In fact, we selected our stimuli based on their unimodal reaction time differences, arguing that stimuli eliciting fast/slow reaction times would result in less/more audio-visual facilitation. This was indeed the case when correcting for overall reaction time differences across subjects (Figure 4). It is interesting to note that despite differences in the neuronal encoding mechanisms for amplitude-modulation (Joris et al., 2003; Wang et al., 2008), and especially the difference between temporal (low ω‘s) and rate (high ω‘s) codes, the inverse effectiveness principle holds true.

## Conclusions

Taken together our data suggest that despite marked differences in unimodal processing humans and monkeys experience similar audio-visual integration through mechanisms that rely on temporal coincidence and inverse effectiveness of the unimodal stimuli. The rhesus macaque may be considered an excellent proxy for the study of neuronal mechanisms underlying multi-sensory integration.

## Author contributions

PB, and RM designed the research; PB, and RM performed the experiments; AVO, contributed materials and reagents; PB analyzed the data; PB, MVW, and AVO discussed the data, PB drafted the manuscript; PB, RM, MVW, and AVO wrote the paper.

### Conflict of interest statement

The authors declare that the research was conducted in the absence of any commercial or financial relationships that could be construed as a potential conflict of interest.

## Abbreviations

LED: light-emitting diode
RT: reaction time
RTP: redundant target paradigm.
